# Impairment in karrikin but not strigolactone sensing enhances root skewing in *Arabidopsis thaliana*

**DOI:** 10.1111/tpj.14233

**Published:** 2019-03-11

**Authors:** Stéphanie M. Swarbreck, Yannick Guerringue, Elsa Matthus, Fiona J. C. Jamieson, Julia M. Davies

**Affiliations:** ^1^ Department of Plant Sciences University of Cambridge Cambridge CB2 3EA UK; ^2^ ENS de Lyon ‐ Site Monod Lyon 69007 France; ^3^ Department of Plant Sciences University of Oxford South Parks Road Oxford OX1 3RB UK

**Keywords:** karrikin, strigolactone, *Arabidopsis thaliana*, waving, root, skewing

## Abstract

Roots form highly complex systems varying in growth direction and branching pattern to forage for nutrients efficiently. Here mutations in the KAI2 (KARRIKIN INSENSITIVE) α/β‐fold hydrolase and the MAX2 (MORE AXILLARY GROWTH 2) F‐box leucine‐rich protein, which together perceive karrikins (smoke‐derived butenolides), caused alteration in root skewing in *Arabidopsis thaliana*. This phenotype was independent of endogenous strigolactones perception by the D14 α/β‐fold hydrolase and MAX2. Thus, KAI2/MAX2 effect on root growth may be through the perception of endogenous KAI2‐ligands (KLs), which have yet to be identified. Upon perception of a ligand, a KAI2/MAX2 complex is formed together with additional target proteins before ubiquitination and degradation through the 26S proteasome. Using a genetic approach, we show that SMAX1 (SUPPRESSOR OF MAX2‐1)/SMXL2 and SMXL6,7,8 (SUPPRESSOR OF MAX2‐1‐LIKE) are also likely degradation targets for the KAI2/MAX2 complex in the context of root skewing. In *A. thaliana* therefore, KAI2 and MAX2 act to limit root skewing, while *kai2*'s gravitropic and mechano‐sensing responses remained largely unaffected. Many proteins are involved in root skewing, and we investigated the link between MAX2 and two members of the SKS/SKU family. Though KLs are yet to be identified in plants, our data support the hypothesis that they are present and can affect root skewing.

## Significance Statement

*Arabidopsis thaliana* mutants deficient in karrikins/KAI2‐ligand (KL) perception display an exaggerated root‐skewing phenotype, which is not readily explained by impairments in their mechano‐sensing and gravitropic responses. Strigolactones (SLs) belong to a recently characterised set of phytohormones. Their role in root skewing in Arabidopsis has been hypothesized, but no evidence supporting a role for SL in influencing root skewing was found here. Rather, results support a role for as‐yet unidentified endogenous KLs.

## Introduction

Roots grow in complex patterns that are highly relevant to their adaptation to different soil conditions and yet very difficult to investigate in this complex medium. *Arabidopsis thaliana* roots grown vertically on solid medium produce specific surface‐dependent growth patterns described as skewing (deviation from vertical) and waving (Roy and Bassham, 2014). Established differences amongst Arabidopsis ecotypes suggest that these patterns may reflect an adaptive response relevant to natural soil conditions (Vaughn and Masson, 2011; Schultz *et al*., 2017).

**Figure 1:**
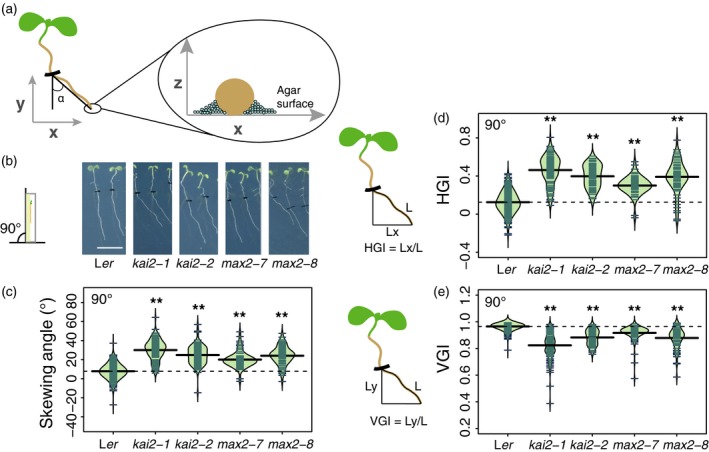
*kai2* and *max2* mutants display an exaggerated rightward root‐skewing phenotype. (a) Arabidopsis roots grown on the surface of agar medium display a root‐skewing pattern that is the result of movement along the *x*‐, *y*‐ and *z*‐axes. (b) Seedlings of *kai2‐1*,* kai2‐2*,* max2‐7* and *max2‐8* displayed an exaggerated rightward skew when grown at 90°. Scale bar: 1 cm. (c) The root‐skewing angle (α) was measured as the deviation from the vertical for plants grown at a 90° angle. (d) The increased root skewing can also be measured as an increase in horizontal growth index (HGI) or (e) a decrease in vertical growth index (VGI). Data for each genotype are displayed as a beanplot with the skewing angle of individual roots shown as dark green horizontal lines, while the mean is represented by a thick black horizontal line. The estimated density of the distribution is illustrated by the shaded colour. The dashed line corresponds to the mean for the wild‐type. Positive values are rightward skews. Significant differences compared with wild‐type (Tukey HSD) are shown: *P < 0.05, **P < 0.01. For each genotype, *n *> 65 in three separate experiments.

Root skewing has been widely reported (Darwin and Darwin, 1880; Migliaccio and Piconese, 2001; Oliva and Dunand, 2007; Roy and Bassham, 2014; Shih *et al*., 2014), but the model describing its mechanism remains complex and incomplete. As Arabidopsis roots grow on the surface of solid agar, they follow the gravitropic vector (Figure 1a). Arabidopsis mutants impaired in the gravitropic response (Okada and Shimura, 1990) show a root‐skewing phenotype. On Earth, gravitropism is one component of the root‐skewing response, while under micro‐gravity as in the International Space Station directional light can also provide a vector directing growth (Paul *et al*., 2012; Roux, 2012). While gravitropism and negative phototropism can essentially be described in two dimensions, a third dimension must also be considered (*z*), which corresponds to the distance away from the growth surface (Figure 1a). This also allows for root movement or circumnutation along the *z*‐axis (Migliaccio and Piconese, 2001; Simmonds *et al*., 2005), and for this movement to be impaired when the roots touch the surface of the solid medium (Thompson and Holbrook, 2004). Arabidopsis mutants deficient in mechano‐sensing, such as *feronia* (Shih *et al*., 2014) or *cml24* (Wang *et al*., 2011), show a root‐skewing phenotype, which supports a role for thigmotropism (change in growth direction in response to mechanical stimulation from surface contact) in root skewing. While root movement in the *z*‐dimension can affect root skewing, the root‐skewing response is measured as the deviation of root growth along the *x*‐axis that can only be seen when roots are grown on a surface. According to the current model, root skewing represents the integrated root tip response to gravity, negative phototropism, circumnutation and thigmotropism. Thus, the root growth patterns generated are dependent on the forces applied at the root tip and the characteristics of the mature roots, such as size and rigidity.

The role of plant hormones in root skewing and waving is poorly understood, but auxins (Okada and Shimura, 1990), ethylene (Buer *et al*., 2000, 2003), cytokinins (Kushwah *et al*., 2011) and brassinosteroids (Lanza *et al*., 2012) are implicated. Little is known of the role of a recently characterised set of phytohormones, strigolactones (SLs; Roy and Bassham, 2014), and related smoked‐derived butenolides, karrikins (KARs; Flematti *et al*., 2015), or the as‐yet unidentified endogenous ligands of the KAI2 (KARRIKIN INSENSITIVE) KAR receptor [KAI2‐ligand (KL); Sun *et al*., 2016]. Given the role of SLs in regulating root system architecture (Ruyter‐Spira *et al*., 2011; Mayzlish‐Gati *et al*., 2012; Rasmussen *et al*., 2012, 2013; Kapulnik and Koltai, 2014; Sun *et al*., 2014, 2015; Jiang *et al*., 2015; Matthys *et al*., 2016) and affecting auxin transport (Crawford *et al*., 2010; Shinohara *et al*., 2013), it would be interesting to test their role in root skewing.

Many elements of the SL perception pathway have been elucidated, and are either shared or related to components of the KAR/KL perception pathway. The current model suggests that SLs bind a related α/β‐fold hydrolase called D14 (Hamiaux *et al*., 2012; Chevalier *et al*., 2014; de Saint Germain *et al*., 2016; Yao *et al*., 2016), while KARs and KLs are perceived by binding the α/β‐fold hydrolase KAI2/D14‐like protein (Waters *et al*., 2012; Bythell‐Douglas *et al*., 2013; Sun *et al*., 2018). D14 can form a complex with MAX2 (MORE AXILLARY GROWTH2), a leucine‐rich repeat F‐box protein (Zhao *et al*., 2015; Yao *et al*., 2016), while physical interaction between KAI2 and MAX2 was demonstrated using yeast two‐hybrid (Toh *et al*., 2014). The KAR‐dependent degradation of KAI2 can also occur independently from MAX2, independently of ubiquitination or the activity of the 26S proteasome (Waters *et al*., 2015a). More recently, heat‐shock‐related proteins have been identified as degradation targets of MAX2 in rice (Jiang *et al*., 2013; Zhou *et al*., 2013) and Arabidopsis (SMXL, SUPPRESSOR OF MAX2‐1‐LIKE; Stanga *et al*., 2013; Soundappan *et al*., 2015; Moturu *et al*., 2018). Thus far, a dichotomy has been proposed with SMAX1 (SUPPRESSOR OF MAX2‐1) suppressing KAR‐related *max2* phenotypes (e.g. germination and hypocotyl elongation), while other members of the SMXL family, namely SMXL6, SMXL7 and SMXL8, suppress SL‐related phenotypes [e.g. shoot branching and lateral root density (LRD); Waters *et al*., 2017]. Additional members SMXL3, 4 and 5 regulate phloem development in a SL‐ and KAR‐independent manner (Wallner *et al*., 2017). While some specificity of SL or KAR/KL signalling is established through the receptors, additional specificity is reinforced through the degradation targets. These have been described not merely as suppressors of signalling but also as growth regulators, the activities of which are modulated via SL or KAR/KL signalling (Jiang *et al*., 2013).

In this study, we asked whether SL and KAR/KL have a role in regulating root skewing. Using mutants impaired in proteins that are likely receptors for these compounds, we showed that while SL has no effect on root skewing, mutants deficient in KAR/KL perception, *max2* and *kai2*, display an enhanced root‐skewing phenotype. We also investigated the mechanism by which KAI2 and MAX2 modulate root skewing.

## Results

### Mutation in *kai2* and *max2* increases root rightward skew

If KLs or KARs were involved in root skewing, then insensitive Arabidopsis mutants would display an aberrant root‐skewing phenotype. Vertically grown *kai2‐1* and *kai2‐2* mutants showed significantly increased rightward root skewing compared with the L*er* wild‐type (α, root tip displacement, viewed from the back of the plate: Figure 1a–c; Tukey HSD, *P *< 0.01). The root‐skewing angle of *kai2‐2* mutant in the Col‐0 background [*kai2‐2* (6x Col‐0)] was also significantly higher than that of the wild‐type (Figure S1a; Tukey HSD, *P *< 0.01). Vertically grown *max2‐7* and *max2‐8* mutants showed a significant increase in rightward root skewing compared with their *Ler* parental wild‐type (Figure 1b,c; Tukey HSD, *P *< 0.01).

Horizontal growth index (HGI; ratio of root tip displacement along the *x*‐axis to root length; Grabov *et al*., 2004; Vaughn and Masson, 2011) was also significantly higher in *kai2‐1*,* kai2‐2*,* max2‐7* and *max2‐8* compared with wild‐type (Figure 1d; Tukey HSD, *P *< 0.01), supporting the skewing angle data and showing increased deviation from vertical by mutant roots. Similarly, the vertical growth index (VGI; ratio of root tip displacement along the *y‐*axis to root length; Grabov *et al*., 2004; Vaughn and Masson, 2011) was significantly smaller for *kai2‐1*,* kai2‐2*,* max2‐7* and *max2‐8* compared with wild‐type (Figure 1e; Tukey HSD, *P *< 0.01). In separate experiments, two complemented *kai2‐2* lines (driven by the native promoter KAI2:KAI2 *kai2‐2*; Waters *et al*., 2015b) showed a significantly decreased root‐skewing angle compared with *kai2‐2* (Figure S1b; Tukey HSD, *P *< 0.01). Overall these data suggest a role for both KAI2 and MAX2 in preventing exaggerated root skewing in Arabidopsis.

### KAI2 and MAX2 affect root skewing and waving on a tilted surface

**Figure 2:**
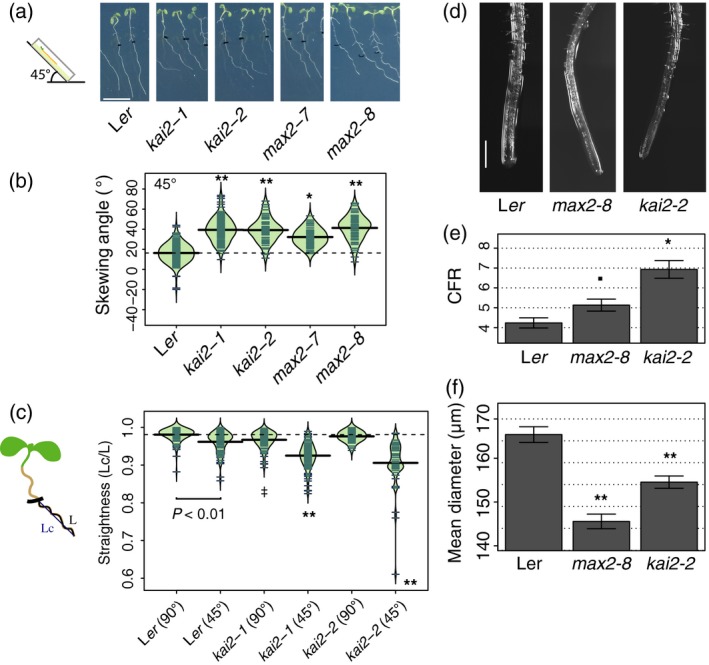
*kai2* and *max2* increased rightward root skewing and cell file rotation (CFR) when placed at 45°. (a) Seedlings of *kai2‐1*,* kai2‐2*,* max2‐7* and *max2‐8* were grown vertically for 6 days, then placed at 45° for 3 days (indicated by a tick on the roots). Scale bar: 1 cm. (b) The root‐skewing angle (α) was measured as the deviation from the vertical for plants grown at a 45° angle for 3 days. (c) The straightness (measured as the ratio of the chord Lc to root length L; Grabov *et al*., 2004; Vaughn and Masson, 2011) of seedling roots from wild‐type, *kai2‐1* and *kai2‐2* decreased when plants were grown at 45° compared with 90° (shown in brackets after genotype). Data for each genotype are displayed as a beanplot with the straightness of individual roots shown as dark green horizontal lines, while the mean is represented by a thick black horizontal line. The estimated density of the distribution is illustrated by the shaded colour. The dashed line corresponds to the mean for the wild‐type. Significant differences compared with wild‐type grown at 45° (Tukey HSD) are shown as **P < 0.01, while comparisons with wild‐type grown at 90° are indicated by ^‡^ *P *< 0.05. For each genotype, *n *> 58 in three separate experiments. (d) Both *max2‐8* and *kai2‐2* mutants show increased CFR, indicating that the root epidermal cells were twisting more compared with those of the wild‐type. CFR was measured as the number of epidermal cells that crossed a 1‐mm line 1.5–2 mm from the root tip. Plants were grown at 45°. (e) Data shown as mean ± se,* n *= 28–42 plants obtained in four separate experiments, *P < 0.05, ^•^ *P *< 0.1. Scale bar: 500 μm. (f) The root diameter of *max2‐7* and *kai2‐2* plants was lower than that of wild‐type. Data shown as mean ± SEM, *n *> 36 per genotype in a total of five experiments, **P < 0.01 (Tukey HSD).

Positioning plates at a 45° angle from the vertical rather than vertically increases the root‐skewing angle. A significant increase in rightward root‐skewing angle was observed here for the L*er* wild‐type grown at a 45° plate angle (Figure 2a,b; anova,* F*_1,510 _= 134.9, *P *< 0.001), while *kai2‐1*,* kai2‐2*,* max2‐7* and *max2‐8* also showed a significantly increased rightward root‐skewing angle compared with L*er* (Figure 2a,b; Tukey HSD, *P *< 0.01, except for *max2‐7* where *P *< 0.05). The increase in mutant root skew relative to wild‐type was maintained at the 45° plate angle compared with growth at 90°, indicating that loss of KAI2 or MAX2 did not affect the mutant's ability to sense and respond to the tilt.

Increased root skewing is often also accompanied by increased root waving (Roy and Bassham, 2014) – a decrease in root straightness calculated as the ratio of the cord over the root length (i.e. straight roots have a ratio of 1 and the lower the ratio the less straight/more wavy the root; Grabov *et al*., 2004; Vaughn and Masson, 2011). Growth on a tilted surface can also decrease straightness (Roy and Bassham, 2014). When grown at 45°, both *kai2‐1* and *kai2‐2* showed a decreased straightness (Figure 2c; Tukey HSD, *P *< 0.01). L*er* was significantly less straight when grown at 45° compared with 90° (Tukey HSD, *P *< 0.01). When grown at a 90° plate angle, *kai2‐1* (Tukey HSD, *P *< 0.05) but not *kai2‐2* (Tukey HSD, n.s.) showed a significantly decreased straightness compared with wild‐type L*er* (Figure 2d). These data show that KAI2 is involved in the negative control of both skewing and waving when plants are grown at an angle, but only skewing when grown vertically.

### KAI2 and MAX2 affect epidermal cell file rotation and root diameter

Although mechanistic models for root skewing vary (Roy and Bassham, 2014), the rotation of epidermal cell files is considered to be an important feature (Sedbrook *et al*., 2002; Oliva and Dunand, 2007; Wang *et al*., 2011). Right‐handed cell file rotation (CFR) was increased in both *kai2‐2* (mean ± se: 6.93 ± 0.44 cell mm^−1^; Tukey HSD, *P *< 0.01) and, to a lesser extent, *max2‐8* (5.13 ± 0.30 cell mm^−1^; Tukey HSD, *P *= 0.08) compared with L*er* wild‐type (4.24 ± 0.25 cell mm^−1^; Figure 2d,e).

Furthermore, the mean root diameter of the mutants was significantly narrower than that of wild‐type (Figure 2f; L*er*: 166.43 ± 1.79 μm; *kai2‐2*: 155.57 ± 1.41 μm; *max2‐8*: 146.59 ± 1.67 μm; Tukey HSD, *P *< 0.001), suggesting that root radial expansion may be restricted.

### *KAI2* and *MAX2* operate through the same genetic pathway

**Figure 3:**
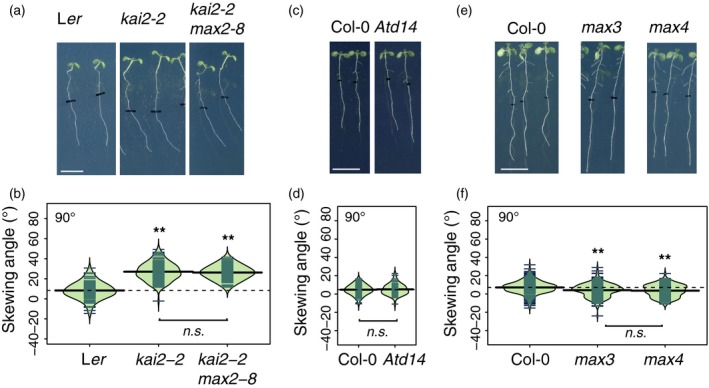
KAI2 and MAX2 regulate root skewing through the same genetic pathway, which does not involve D14. (a, b) Seedlings for the double‐mutant *kai2‐2 max2‐8* showed no further increase in root‐skewing angle compared with *kai2‐2*. Scale bar: 1 cm. Data for each genotype are displayed as a beanplot with the skewing angle of individual roots shown as dark green horizontal lines, while the mean is represented by a thick black horizontal line. The estimated density of the distribution is illustrated by the shaded colour. The dashed line corresponds to the mean for the wild‐type. **Indicates significant difference compared with wild‐type (Tukey HSD, *P *< 0.01). For each genotype, *n *> 66 in five separate experiments. (c) Seedlings for the strigolactone (SL)‐insensitive mutant *Atd14* showed no increased rightward root skewing, and the measured skewing angle was not significantly different from that of the wild‐type (d). For each genotype, *n *> 73 from three experiments. (e, f) Seedlings for the SL synthesis mutants *max3* and *max4* show a slight decrease in rightward root skewing, *n *> 142 from three experiments.

Genetic studies of the elongated hypocotyl phenotypes (Waters *et al*., 2012) have suggested that KAI2 and MAX2 are in the same signalling pathway, and physical interaction between these two proteins has been demonstrated in a yeast two‐hybrid assay (Toh *et al*., 2014). Here we use a similar genetic approach to show that the double‐mutant *kai2‐2 max2‐8* has a significantly increased rightward root skew compared with wild‐type (Figure 3a,b; Tukey HSD, *P *< 0.01), which was not significantly different from that of *kai2‐2* (Figure 3a,b; Tukey HSD, n.s.). That the skewing angle of the *kai2‐2 max2‐8* double‐mutant was not greater than that of the *kai2‐2* single‐mutant suggests that KAI2 and MAX2 operate in the same genetic pathway.

### Karrikin reduces root skewing, but is a poor analogue of KAI2‐ligand

The data demonstrate that in Arabidopsis an impairment in KAR/KL perception leads to greater rightward root skewing. This suggests that perhaps the abundance of KAR or KL in the roots may affect root skewing, and the hypothesis that an increased availability of KL or its analogue KAR_2_ might compensate for a lowered sensitivity of the system and decrease the rightward root skewing. In the absence of purified and identified KL compounds, the effect of KAR on root skewing was tested using the potent KAR_2_ (Nelson *et al*., 2009; Waters *et al*., 2015a). There was a significant effect of KAR_2_ in reducing rightward root skewing of L*er* wild‐type plants with concentrations of 5 and 10 μm (Figure S2a; Tukey HSD, *P *< 0.01). However, a significant inhibitory effect on primary root elongation of L*er* plants was evident at 10 μm KAR_2_ (Figure S2b; Tukey HSD, *P *< 0.01).

The presence of 2.5 and 5 μm KAR_2_ in the medium also significantly decreased the root‐skewing angle of *kai2‐2* (Figure S2b; Tukey HSD, *P *< 0.01). The KAI2‐independent effect of KAR_2_ on root skewing may also be linked to reduced root elongation, as this was significantly lower in the presence of 5 μm KAR_2_ (Figure S2b; Tukey HSD, *P *< 0.01), but not at 2.5 μm (Figure S2b; Tukey HSD, n.s.). Similarly, the presence of 5 μm KAR_2_ in the medium significantly decreased the root‐skewing angle of *max2‐8* (Figure S2e; Tukey HSD, *P *< 0.01) as well as primary root elongation (Figure S2f; Tukey HSD, *P *< 0.01). A negative effect of KAR_1_ on rightward root skewing (Figure S2g) could also be measured in *kai2‐2* and *max2‐8* plants, while L*er* plants remained insensitive. KAR could significantly reduce rightward root skewing; however, this may be an unspecific effect as *kai2* plants also responded and at lower KAR concentrations compared with L*er* plants.

### Strigolactones do not affect root skewing

Given the root‐skewing phenotype of *max2* mutants, and the role of MAX2 in SL perception, we also investigated the role of SL in root skewing. To demonstrate a role for SL in regulating root skewing, we looked for evidence for a phenotype in SL perception (*d14*) and synthesis (*max3*,* max4*) mutants. *Max3* and *max4* mutants are impaired in carotenoid cleavage dioxygenase 7 and 8, respectively, key enzymes involved in SL synthesis (Sorefan *et al*., 2003; Booker *et al*., 2004; Schwartz *et al*., 2004). We also report here the effect of GR24, a widely used analogue of SL. Under our growth conditions, we have found no evidence supporting a role for SL in regulating root skewing. *d14* mutants that are insensitive to SL but not KAR (Waters *et al*., 2012) showed no significant increase in root skewing compared with wild‐type (Figure 3c,d; anova, n.s.). In addition, both *max3* and *max4* show no significant increase in root‐skewing angle, rather they showed a small but significant reduction in root‐skewing angle compared with the wild‐type Col‐0 (Tukey HSD, *P *< 0.01, for both *max3* and *max4*).

A racemic mix of GR24 (GR24_rac_) that was shown to regulate root growth (Kapulnik *et al*., 2011; Ruyter‐Spira *et al*., 2011; Rasmussen *et al*., 2012) and that can also be perceived by KAI2 (Scaffidi *et al*., 2014; Waters *et al*., 2015a) was tested at 1 and 5 μm, as greater concentrations tended to have a toxicity effect on root growth (Ruyter‐Spira *et al*., 2011). Treatment with GR24_rac_ led to a small increase in rightward root skewing in L*er* plants at 1 μm (Figure S3a; Tukey HSD, *P *< 0.05) but not at 5 μm GR24_rac_ (Tukey HSD, n.s.), whereas only *kai2‐2* and not *kai2‐1* responded with a decrease in rightward root skewing at 5 μm GR24_rac_ (Tukey HSD, *P *< 0.05). There was no significant effect of 1 or 5 μm GR24_rac_ on the root skewing of Col‐0 plants (Figure S3b; anova,* F*_2,261 _= 1.26, n.s.), whereas *max2*, but not *d14* (anova,* F*_2,184 _= 1.31, n.s.), showed a small but significant increase in root‐skewing angle under 1 μm GR24_rac_ (Tukey HSD, *P < *0.05) but not 5 μm GR24_rac_ (Tukey HSD, n.s.).

GR24_rac_ chemically complements SL‐deficient mutants, *max3* and *max4* (Ruyter‐Spira *et al*., 2011; Rasmussen *et al*., 2012). If SL could affect root skewing, *max3* and *max4* mutants (which showed a reduced root‐skewing phenotype compared with wild‐type; Figure 3e,f) should show a slight increase in rightward root skewing in the presence of GR24. However, both mutants showed a further decrease in the skewing angle, in the presence of 5 μm GR24 (Tukey HSD, *P *< 0.01).

Moreover, the root skewing of mutants deficient in DLK2 (D14‐LIKE 2) proteins (Waters *et al*., 2012) was not significantly different to wild‐type (Figure S1c; Tukey HSD, n.s.). As the DLK2 protein is related to both KAI2 and D14, overall these data demonstrate a specific role for KAI2 and MAX2 in modulating root skewing and thus implicate KL/KAR, and not SL, sensing through these proteins.

### MAX2 effect on root skewing requires SMAX1, SMXL2 and SMXL6,7,8

**Figure 4:**
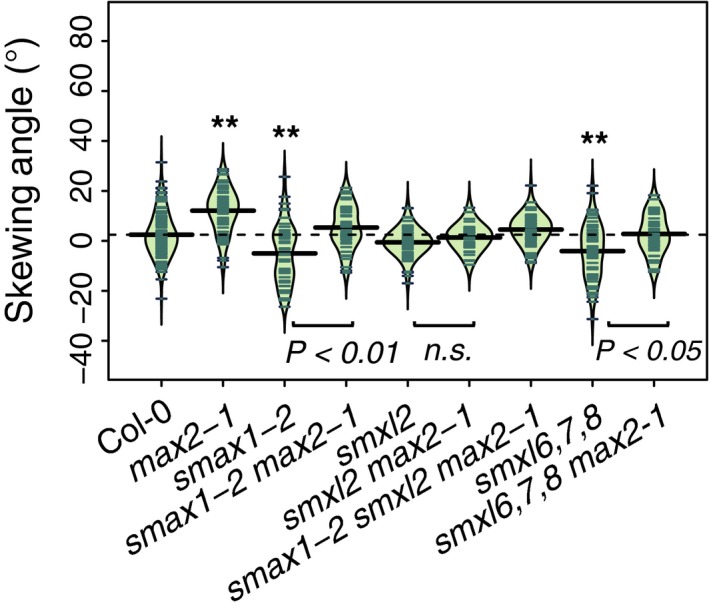
MAX2 effect on root skewing requires SMAX1, SMXL2 and SMXL6,7,8. Root‐skewing phenotypes of Col‐0, *max2‐1*,* smax2‐1*,* smax2‐1 max2‐1*,* smxl2*,* smxl2 max2‐1*,* smax1‐2 smxl2 max2‐1*,* smxl6,7,8* and *smxl6,7,8 max2‐1* while grown at 90°. Data for each genotype are displayed as a beanplot with the skewing angle of individual roots shown as dark green horizontal lines, while the mean is represented by a thick black horizontal line. The estimated density of the distribution is illustrated by the shaded colour. The dashed line corresponds to the mean for the wild‐type. Significant differences compared with wild‐type (Tukey HSD) are shown: *P < 0.05 and **P < 0.01. For each genotype, *n *> 38 from three experiments.

The effect of MAX2 degradation targets, SMAX1 (SUPPRESSOR OF MAX2‐1) and SMXLs (SUPPRESSOR OF MAX2‐1‐LIKE, Stanga *et al*., 2013), on root skewing was examined, thus testing the hypothesis that the MAX2‐dependent regulation of protein abundance for members of the SMAX/SMXL family is relevant to the root‐skewing phenotype. The current mechanistic model for Arabidopsis is that SMAX1 and SMXL2 are important for the KL part of the signalling pathway, whereas SMXL6,7,8 are more relevant to the SL part of the pathway (Soundappan *et al*., 2015). Here we report that there was no significant difference between Col‐0 and *max2 smax1‐2* (Tukey HSD, n.s.), Col‐0 and *max2‐1 smxl2* (Tukey HSD, n.s.), Col‐0 and *smax1‐2 smxl2 max2‐1* (Tukey HSD, n.s.) or Col‐0 and *smxl6,7,8 max2‐1* (Tukey HSD, n.s.), thus it seems that the absence of SMAX1, SMXL2 or SMXL6,7,8 suppresses the *max2* phenotype. Interestingly, both *smax1‐2* and *smxl6,7,8* mutants, but not *smxl2* (Tukey HSD, n.s.), showed a significant decrease in root‐skewing phenotype compared with wild‐type (Figure 4a,b; Tukey HSD, *P *< 0.01), thus reinforcing the idea that the abundance of these proteins affects root skewing. Interestingly, while SMXL3, 4 and 5 are central regulators for phloem formation, and mutants deficient in at least two members of this subclade display a short and thin root phenotype, no skewing phenotype was reported (Wallner *et al*., 2017).

### *kai2* and *max2* can support a near‐normal mechano‐sensing response

**Figure 5:**
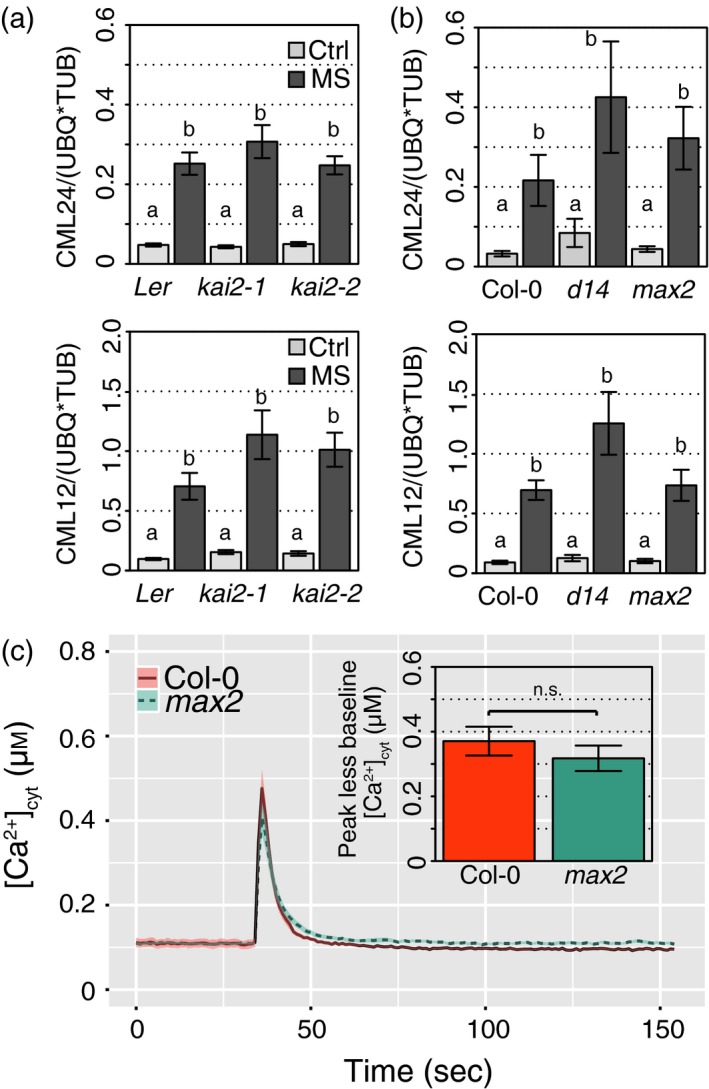
*kai2*,* max2*,* d14* mutants support a near‐normal response to mechano‐stimulus. Karrikin (KAR)‐ and strigolactone (SL)‐insensitive mutants showed a normal upregulation of touch response genes, in response to mechanical stimulation. Nine‐day‐old seedlings of wild‐type and mutants (a) *kai2‐1* and *kai2‐2*, and (b) *d14*,* max2‐1* were mechanically stimulated (MS) for 30 sec, then collected 30 min later for transcript analysis of touch‐sensitive genes *CML12* and *CML24,* relative to housekeeping genes *Tubulin 4* and *Ubiquitin 10*. The means of six–nine replicates from three independent experiments are shown, each replicate based on the RNA extracted from roots of 30–40 seedlings. Data are shown as mean ± se, letters indicate significant differences (Tukey HSD, *P *< 0.01). (c) Mechano‐stimulated [Ca^2+^]_cyt_ increase in *max2* root tips. Individual excised root tips of Col‐0 and *max2* expressing (apo)aequorin as a [Ca^2+^]_cyt_ reporter were mechanically stimulated by addition of buffer at 35 sec. The mean ± SEM of 40–67 roots in five independent trials are shown. Inset: mean ± SEM maximal [Ca^2+^]_cyt_ increment in response to stimulus (peak response minus baseline).

The growth responses of the *kai2* mutants on tilted plates suggested that the mutation does not affect the root tip's ability to sense the increased mechanical impedance afforded by the inclined growth medium. Rather, that the *kai2* mutants have an exaggerated root skew when grown on a tilted surface suggests that downstream responses are impaired. To test for a role for KAI2 in mechano‐sensing responses, seedlings were subjected to mechanical stress prior to determination of root transcript levels of *CML12* and *CML24* (CALMODULIN‐LIKE PROTEIN; Figure 5a). These transcripts are known to increase upon mechanical stimulation (Braam and Davis, 1990). These tests also addressed *max2* and *d14* in the Col‐0 background (Figure 5b). Mechanical stimulation caused significant upregulation of *CML12* and *CML24* transcript in roots of all genotypes tested (anova,* P *< 0.01), but no mutants responded significantly differently to the wild‐type. Thus, the data suggest that root transcriptional mechano‐responsiveness is not drastically altered in either KL‐ or SL‐insensitive mutants.

As a final test for alteration in mechano‐sensing and response, *max2* (as the common lesion in KL‐ and SL‐pathways) was transformed to express (apo)aequorin as a reporter of cytosolic free Ca^2+^ ([Ca^2+^]_cyt_). [Ca^2+^]_cyt_ increases transiently in response to mechano‐stimulation, acting as a second messenger (Knight *et al*., 1991; Shih *et al*., 2014). There was no significant difference between baseline level pre‐injection and post‐injection for Col‐0 (*t*‐test, n.s.) or *max2* (*t*‐test, n.s.). There was no significant difference in the amplitude of the touch‐induced peak increase in [Ca^2+^]_cyt_ between genotypes (Figure 5c; *t*‐test, n.s.). However, the total Ca^2+^ mobilised over the recording period (excluding the discharge) for *max2* (33.99 ± 0.57 μm) was significantly higher than that for Col‐0 (29.91 ± 0.49 μm;* t*‐test, *P *< 0.01). This is in contrast to the *feronia* plasma membrane receptor‐like kinase mutant that fails to support normal touch‐induced [Ca^2+^]_cyt_ elevation but, in common with *max2* and *kai2*, has rightward‐skewing roots (Shih *et al*., 2014). Therefore, there is no clear link between calcium handling and root‐skewing phenotype of *max2*.

### *kai2* but not *max2* has a slower early gravitropic response

**Figure 6:**
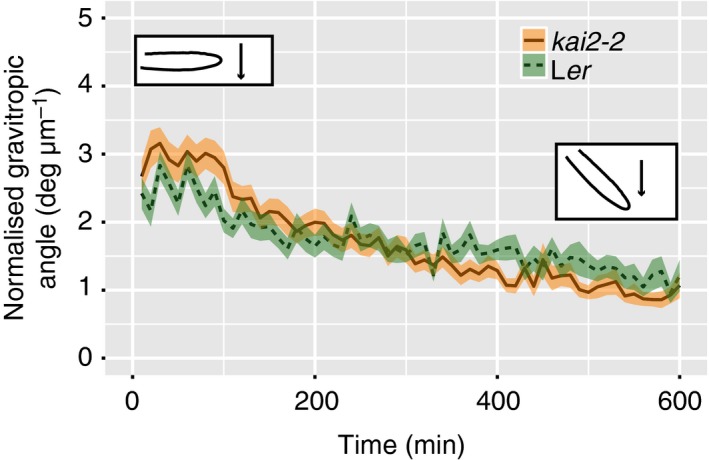
Gravitropic response of *kai2* is slower than that of wild‐type. The tip orientation of roots from wild‐type and *kai2‐2* was recorded every 10 min and for 10 h after a change in gravitropic orientation. The change in tip orientation was normalised to the tip displacement to take into account differences in growth rate between genotypes. Data are shown as mean ± se,* n *= 16–22 plants obtained in five experiments.

Agravitropic mutants can also show an increased root skewing (Okada and Shimura, 1990). To investigate whether an aberrant gravitropic response of *kai2‐2* plants contributed to their skewing phenotype, root tip orientation was monitored every 10 min after gravistimulation for 10 h. Both *kai2‐2* and wild‐type responded significantly with a change in tip orientation over time (Figure 6; anova, *F*_1,4022 _= 46.8, *P *< 0.01). Comparisons of the responses (normalised for elongation rate) using anova showed that there was a significant interaction between time and genotype (anova,* F*_1,4022 _= 40.9, *P *< 0.01), indicating a difference in gravitropic response between genotypes. *kai2‐2* has a dampened initial response as root tip angle started to decrease later than L*er*. After 100 min, the angle of *kai2‐2* was significantly higher than that of L*er* (anova,* F*_1,64 _= 4.4, *P *< 0.01), but at 600 min there was no significant difference (anova,* F*_1,64 _= 0.24, n.s.). Overall, the difference in gravitropic response between *kai2‐2* and L*er* may be a small contributory factor to root skewing, but occurring only in the early stages of the response.

The gravitropic response of SL synthesis and perception mutants was also monitored (Figure S4). All genotypes responded to the shift in gravitropic angle with a change in tip orientation over time (anova, *F*_1,2188 _= 1343.1, *P *< 0.01). There was a significant difference amongst genotypes (anova, *F*_3,2188 _= 30.1, *P *< 0.01), with SL‐insensitive and synthesis mutants showing earlier change in normalised gravitropic angle, compared with wild‐type. Although both *kai2* and *max2* were impaired in their initial gravitropic responses, their contrasting responses (delayed and earlier, respectively) are inconsistent with both skewing rightward.

### *sks3* suppresses *max2* root skewing but not lateral root density phenotype

**Figure 7:**
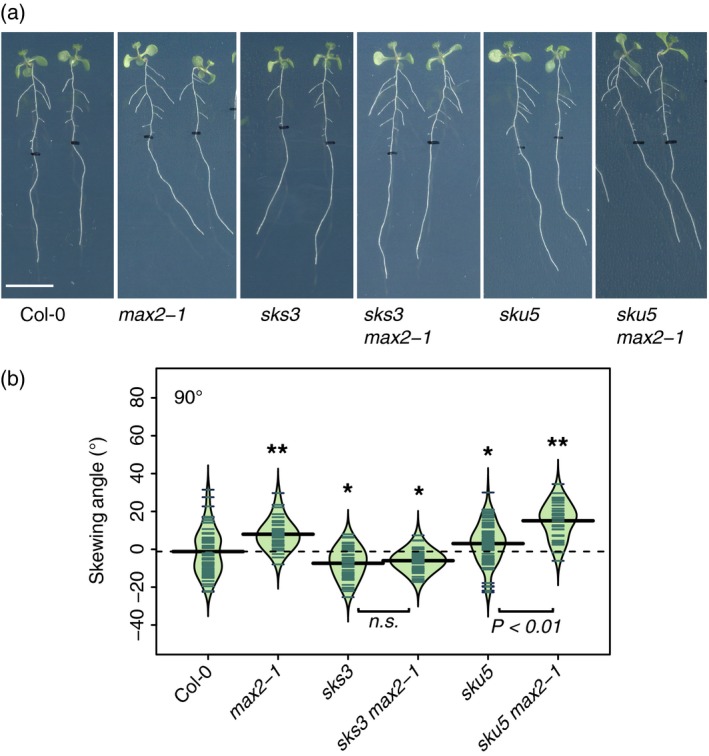
MAX2 regulation of root skewing involves SKS3. (a) Seedlings of Col‐0, *max2‐1*,* sks3*,* sks3/max2‐1*,* sku5*,* sku5/max2‐1* mutants grown at 90°. Scale bar: 1 cm. (b) Data for each genotype are displayed as a beanplot with the skewing angle of individual roots shown as dark green horizontal lines, while the mean is represented by a thick black horizontal line. The estimated density of the distribution is illustrated by the shaded colour. The dashed line corresponds to the mean for the wild‐type. *Indicates a significant difference compared with wild‐type (Tukey HSD, *P *< 0.05). For each genotype, *n *> 34 in three separate experiments.

Similarly to the *kai2* and *max2* mutants, mutant plants deficient in the SKU5 protein that is linked to the plasma membrane by a glycosylphosphatidylinositol anchor also showed an increased rightward root‐skewing phenotype, increased CFR with no change in gravitropic response (Sedbrook *et al*., 2002). In our experiments, *sku5* also displayed a rightward skew when grown vertically that was significantly greater than the wild‐type (Figure 7a,b; Tukey HSD, *P *< 0.05). We further investigated the phenotype of a related mutant, deficient in *sks3* (*sku5 similar 3*), as well as multiple mutants deficient in members of the SKS/SKU family (Zhou, 2013). The *sks3* mutant skewed to the left (Tukey HSD, *P *< 0.05). Interestingly, the root‐skewing phenotype of *sks3* was maintained even in the absence of MAX2 (comparison *sks3*:* sks3 max2‐1*, Tukey HSD, n.s.), suggesting that *sks3* completely suppresses the *max2* phenotype. The skewing angle of *sku5 max2* was not significantly higher than that of *max2* (Tukey HSD, n.s.). *sks3* and *sku5* do not suppress the high LRD phenotype (Figure S5) or the decreased germination rate of *max2* mutants (Figure S5). These data suggest that the abundance of SKS3 protein may itself affect root skewing, and support the previous report of a root‐skewing phenotype in *sku5*.

The cellular localisation of *SKS3* (At5g48450) and *SKU5* (At4g12420) was surveyed using available online high‐resolution root expression patterns (Brady *et al*., 2007). *SKS3* is expressed in the cortex in the meristematic zone (Figure S6a), and at a lower level compared with *SKU5* (Figure S6c,f). *SKU5* mRNA could be detected in the endodermis in the meristematic and elongation zones, as well as the vasculature, particularly procambium of the meristematic zone (Figure S6d). *KAI2* (At4g37470) transcripts occur in the vasculature (procambium) throughout the root, *MAX2* (At2g42620) is also expressed in the vasculature but in the phloem companion cells and mostly in the elongation and maturation zones (Figure S7; Winter *et al*., 2007). Besides the overlap between *SKU5* and *KAI2* in the procambium in the meristematic zone, there is little overlap between *KAI2*,* MAX2*,* SKU5* and *SKS3* expression patterns.

## Discussion

The characterisation of phenotypes for *kai2* and *max2* mutants that are unrelated to the presence of smoke, as well as the presence of these receptors in non‐fire following plants have led to the hypothesis that endogenous KLs are present in plants and act as phytohormones (Conn and Nelson, 2016). Evidence supporting their presence in the water‐soluble fraction of Arabidopsis shoot extract has now been reported (Sun *et al*., 2016). Here we report evidence demonstrating a role for KAI2 and MAX2 in preventing exaggerated root skewing in Arabidopsis, thus providing additional support for a hypothetical endogenous KL.

Phytohormones such as auxins, ethylene and cytokinins have been implicated in the regulation of root skewing, but thus far the role of SLs has remained unknown (Roy and Bassham, 2014). In Arabidopsis, SLs are primarily produced in the roots and transported in the xylem (Goldwasser *et al*., 2008; Kohlen *et al*., 2011), though some SL production could be shown in the shoots (for review, see Al‐Babili and Bouwmeester, 2015). They have been shown to regulate LRD, primary root elongation, root hair elongation and adventitious root growth (Koltai *et al*., 2009, 2010; Ruyter‐Spira *et al*., 2011; Rasmussen *et al*., 2012, 2013; Shinohara *et al*., 2013; Kapulnik and Koltai, 2014), but root skewing or waving phenotypes have not been reported. We investigated the root‐skewing phenotype of SL mutants, and found no evidence supporting a role for endogenous SLs in regulating root skewing. While *max2* mutants displayed an increased root‐skewing phenotype, this is not the case for *d14*, and we can attribute the phenotype of *max2* to its role in KAR/KL sensing. Mutants deficient in SL synthesis (*max3*,* max4*) show a reduced root‐skewing angle compared with wild‐type, but this is not chemically complemented by providing exogenous GR24_rac_. Thus, we propose that SL has no role in regulating root skewing in Arabidopsis, at least under our growth conditions.

Mutants have proved useful in identifying new components of the machinery regulating root skewing in Arabidopsis. Here, the increased root‐skewing phenotype of *kai2* and *max2* suggests that both KAI2 and MAX2 negatively regulate root skewing. Because these two proteins are involved in the perception of KAR/KL, this provides evidence supporting a role for KL in regulating root skewing, though it is possible that the effects of KAI2 and MAX2 may be ligand independent. These two proteins have been implicated in promoting germination, hypocotyl elongation, light response, reducing hyponasty, establishing arbuscular mycorrhizal fungal (AMF) symbiosis, affecting leaf morphology and drought resistance (Nelson *et al*., 2011; Sun and Ni, 2011; Waters *et al*., 2012; Stanga *et al*., 2013; Gutjahr *et al*., 2015; Li *et al*., 2017; Lee *et al*., 2018). Root‐related phenotypes reported for any mutant deficient in KAI2 include the absence of AMF symbiosis in rice (Gutjahr *et al*., 2015) and a lower large LRD in rice (Chiu *et al*., 2017). The AMF symbiosis seems to be impaired in the early stages of the interactions as no physical contact between plant and fungus or gene expression changes generally triggered by fungal signals could be found. Our data provide additional evidence of the functionality of KAI2 and MAX2 proteins in roots. In common with the symbiosis phenotype, it is currently difficult to resolve a phenotype that appears to relate to a response at the epidermal level (e.g. change in CFR in skewing) with the vascular localisation of *max2* and *kai2* expression, although the KAI2 and MAX2 protein localisation may differ from that of the mRNA. Interestingly, *KAI2* light‐induced expression in the short term is *hy5*‐dependent (Waters and Smith, 2013), with the HY5 transcription factor binding to both a C/G‐box and a G‐box in the *KAI2* promoter (Sun and Ni, 2011). The *hy5* mutant was shown to have a root‐skewing phenotype (Oyama *et al*., 1997). Though the KAI2 and HY5 act largely independently to regulate hypocotyl length (Waters and Smith, 2013), it is unclear whether they might operate in the same pathway to regulate root skewing.

In the absence of endogenous purified KL compounds, KARs have been used as analogues to KLs. For phenotypes such as elongated hypocotyls or increased seed dormancy (Waters *et al*., 2012), KAR_2_ acts as a good synthetic analogue for KL (Conn and Nelson, 2016). However, this is not the case for root skewing. A high concentration of KAR_2_ or KAR_1_ was necessary to induce a root‐skewing phenotype, and this effect was KAI2 and MAX2‐independent. Interestingly, KAR_2_ also failed to induce significant changes in gene expression in rice roots (Gutjahr *et al*., 2015). Many SL compounds, which may be structurally related to KL, have been purified thus far (Bouwmeester *et al*., 2007), and perhaps there is also some structural diversity amongst KL compounds. Given that neither KAR nor GR24_rac_ can affect the root‐skewing phenotype in a MAX2‐ and KAI2‐dependent way, we argue that they are poor KL analogues with regards to the regulation of root skewing, and that they have limited use in the study of KAI2/MAX2 regulation of this phenotype.

In common with the perception systems for phytohormones such as auxins, giberellins or jasmonate (Gray *et al*., 2001; Dill *et al*., 2004; Thines *et al*., 2007), the current model proposes that SL and KAR/KL are perceived by binding to an α/β hydrolase receptor (D14 or KAI2), which is then recruited by a SCF^MAX2^ complex that also includes proteins targeted for ubiquitination and degradation through the 26S proteasome. In Arabidopsis, degradation targets for the D14/MAX2 complex were first identified through a genetic screen for suppressors of the *max2*'s low germination phenotype (Stanga *et al*., 2013). This study identified SMAX1 (Stanga *et al*., 2013), with additional members of the SMAX/SMXL family subsequently identified due to sequence similarity (Soundappan *et al*., 2015). Furthermore, the MAX2‐ and D14‐dependent degradation of SMXL7 upon treatment with GR24 demonstrated that this protein was effectively a target for degradation in Arabidopsis (Soundappan *et al*., 2015). In rice, D53 protein (orthologue to SMAX1) was identified as a suppressor of the SL pathway (Jiang *et al*., 2013; Zhou *et al*., 2013). Detailed analyses demonstrated the degradation of D53 in a GR24_rac_‐ as well as a D3‐ and D14‐dependent manner. Polyubiquination of D53 was also demonstrated upon treatment with GR24_rac_ but not KAR_1_. Demonstrating the SMAX/SMXL degradation or interaction with MAX2 or D14 has relied a lot (but not exclusively) on the use of GR24_rac_ as ligand. Interestingly, thus far the KAR/KL‐dependent degradation of members of the SMAX/SMXL family has not been shown yet. Perhaps it is that the KARs currently used are poor mimics of the endogenous ligand that could trigger the complex assembly, ubiquitination and degradation of the target proteins, and that the unavailability of such endogenous ligands is impeding these experiments. It is also possible that the KAR/KL signalling pathway operates following a different model.

The current model proposes a dichotomy between SMAX1‐KAI2‐KAR/KL‐, SMXL6,7,8‐ and D14‐SL‐signaling pathways (Soundappan *et al*., 2015), while SMXL3, 4 and 5 are regulated independently from KAR and SL (Wallner *et al*., 2017). However, our data do not support this idea, and support a role for MAX2 in regulating root skewing in a D14‐independent manner through SMXL6,7,8 as well as SMAX1/SMXL2. Thus, the dichotomy in terms of degradation targets may hold only for some phenotypes. Much may depend on the spatial localisation of proteins. SMAX1 is expressed in the root cap, while SMXL6, 7 and 8 are also present in the vasculature of mature roots (Soundappan *et al*., 2015). *KAI2* expression could be found preferentially in the vasculature (Brady *et al*., 2007) potentially favouring interaction with SMXL6, 7 or 8.

It is unclear how changes in abundance of SMAX/SMXL proteins may affect root skewing, besides targeted changes in gene expression through their interaction with the TOPLESS/TOPLESS‐RELATED family of transcriptional co‐repressors (Jiang *et al*., 2013; Soundappan *et al*., 2015). Furthermore, it is likely that members of the SMAX/SMXL family are not the only proteins targeted for degradation through MAX2. Out of 117 proteins shown to be differentially abundant in *max2* roots compared with wild‐type, the abundance of only nine of these was different between *max2* and wild‐type in the presence of GR24_rac_ (Walton *et al*., 2016). This suggests that MAX2 can lead to change in the abundance of many more proteins beyond SL signalling. Thus, we also considered additional proteins that may regulate the root‐skewing phenotype. Members of the SKS/SKU family, especially SKU5 and SKU6, have been shown to have a root‐skewing phenotype, though the *sku6/spr1‐6* mutant also showed a twisted petiole phenotype that was not noted in *kai2* or *max2* mutants (Sedbrook *et al*., 2002; Sedbrook, 2004). Given the proposed role for the KAI2/MAX2 in targeting protein for degradation and given the phenotypes of multiple mutants (Figure 7), it is fair to hypothesise that SKS3 may be a target for degradation via the KAI2/MAX2 complex in the context of the root‐skewing phenotype. Though genetic data have been used in the past to demonstrate such links (Stanga *et al*., 2013; Soundappan *et al*., 2015), further data supporting a protein–protein interaction or evidence of degradation would be necessary. As discussed above, such experiments may be difficult to execute in the absence of an appropriate ligand. Interestingly, SKU5 could be detected and remain unchanged in the root proteome of *max2* compared with Col‐0 plants (Walton *et al*., 2016). SKS3 protein could not be detected perhaps because it is expressed at much lower levels, compared with SKU5 (Figure S6). Thus far, germination, hypocotyl length and leaf shape have been standard phenotypes used to investigate the KAR/KL signalling pathway. We propose that the root‐skewing phenotype may be useful to identify new proteins targeted for degradation and gain new information on the signalling pathway for KAR/KL.

Here we demonstrated that functional KAI2 and MAX2 proteins could prevent an exaggerated root skewing. Given the known role for KAI2 and MAX2 in plants, it is currently difficult to provide a functional explanation for this phenotype. Furthermore, the mechanism by which KAI2 and MAX2 regulates root skewing remains elusive. We found no evidence supporting a role for KAI2 and MAX2 in regulating the root touch‐dependent upregulation of *CML12* and *CML24,* suggesting that the mechano‐sensitive transcriptional response may be unaltered. The mechano‐stimulated [Ca^2+^]_cyt_ response appears overall similar in *max2* compared with wild‐type, and this does not correlate well with *feronia*'s impaired mechano‐stimulated [Ca^2+^]_cyt_ response but similar rightward skew (Shih *et al*., 2014). In addition, the *kai2* and *max2* mutants show opposite response to change in the gravitropic vector. Thus, an altered gravitropic response does not help explain the root‐skewing phenotype. The enhanced CFR and lower root diameter measured in both *max2* and *kai2* mutants may lead us to an explanation. Perhaps thinner roots encounter less friction with the underlying medium, leading to greater rotation and skewing. However, further work is needed to firmly establish the mechanism of root skewing and the role of KAI2 and MAX2.

The link established here between MAX2 and the SKU/SKS family suggested an interesting possibility that the *max2* skewing phenotype is linked to cell wall modification or integrity. A case supporting a role for cell wall in affecting root skewing is already well established in the literature, with root‐skewing mutants showing clear cell wall phenotype (Nakashima *et al*., 2013; Lim *et al*., 2014; Roy and Bassham, 2014; Van der Does *et al*., 2017) as well as microarrays of roots showing different skewing angles (Vaughn and Masson, 2011; Schultz *et al*., 2017). For example, amongst the 11 highly probable skew gene candidates identified in Arabidopsis roots using microarrays, three were associated to the cell wall either because of their physical location (*PAP24*), or because of their role in cell wall integrity (*DIN2*) or formation (*MIOX4*; Schultz *et al*., 2017).

Several lines of evidence suggest that KL and KAR affect cell wall composition, though the sugar composition of *max2* and *kai2* mutants cell wall appears normal (J. Mortimer, personal communication). Amongst the 133 genes that are differentially regulated 24 h post‐imbibition with 1 μm KAR1, 11 relate to the cell wall, and genes belonging to the ‘plant‐cell type cell wall’ category of the GO cellular components were significantly enriched in the set of genes regulated by KAR1 (Nelson *et al*., 2010). Genes involved in cell wall organisation were also significantly enriched within a set of upregulated genes in the shoots of *kai2‐2* compared with wild‐type (27 genes out of 680 significantly upregulated in *kai2‐2*; Li *et al*., 2017). Interestingly, two fatty acid reductase genes (At3g44560, At5g22500) that are involved in suberin biosynthesis were upregulated in both *kai2* and *max2* mutants compared with wild‐type (Li *et al*., 2017). In addition, metabolomic analyses showed reduced levels of phenylpropanoid contributing to lignin composition (including ρ‐coumaric acids and ferulic acids) in *max2* roots compared with wild‐type roots under control conditions (Walton *et al*., 2016). These are also good indicators of lower levels of cutin monomer, which signals in the AMF‐root symbiosis (Wang *et al*. 2012). Furthermore, transmission electron microscopy images of *kai2* mutants revealed a thinner cuticle, while wild‐type and over‐expressor lines showed a thicker cuticle (Li *et al*., 2017). Thus, an altered cell wall would fit with the impairment in the early events leading to the establishment of KAI2‐dependent AMF symbiosis in host species (Gutjahr *et al*., 2015), and could feasibly influence root skewing and waving.

## Experimental Procedures

### Plant material and growth conditions

Wild‐type Arabidopsis seeds Columbia‐0 (Col‐0) and Landsberg *erecta* (L*er*) were the parental backgrounds for the mutants tested. Seeds for *max2* (*max2‐1*; Stirnberg *et al*., 2002), *max3* (*max3‐9*; Booker *et al*., 2004), *max4* (*max4‐1*; Sorefan *et al*., 2003), *Atd14* (*Atd14‐1*; Waters *et al*., 2012), as well as *sku5* (salk_070056), *sks3* (salk_0677925), *sks3 max2*‐1 and *sku5 max2‐1* were provided by Prof. Dame Ottoline Leyser (SLCU). Seeds for *max2‐7*,* max2‐8*,* kai2‐1*,* kai2‐2*,* dlk2‐1*,* dlk2‐2*,* dlk2‐3* and KAI2:KAI2 (*kai2‐2*) were a gift from Dr Mark Waters (University of Western Australia; Waters *et al*., 2012, 2015b). The *Kai2‐2* allele was backcrossed six times to Col‐0 [*kai2‐2* (6 × Col‐0)] and was a gift from Dr Mark Waters. Seeds were surface‐sterilised by treatment with 70% (v/v) ethanol, followed by a rinse with sterile distilled water, then incubation in 10% (v/v) sodium hypochlorite, 0.05% (v/v) Triton X‐100 for 5 min at 20°C with shaking (1250 rpm). After a further five washes with sterile distilled water, seeds were placed on the surface of 0.8% (w/v) agar (BD, UK) supplemented with ½ MS (Murashige and Skoog including vitamins, pH 5.6; Duchefa, The Netherlands). Arabidopsis seeds were stratified in the dark for 2 days at 4°C, before transfer to a growth cabinet under controlled conditions at 23°C, 16 h light: 8 h dark, and 80 μmol m^−2 ^sec^−1^ irradiance. Growth plates were vertical unless stated otherwise.

### Root‐skewing assay

After 9 days, images were taken by scanning plates from the back (i.e. roots were imaged through the agar) using a flat‐bed scanner (300 dpi), and root‐skewing angles were measured in ImageJ (Schneider *et al*., 2012) using the angle tool. NeuronJ (Meijering *et al*., 2004) was used to record the *x*‐ and *y*‐coordinates of the root tips and a marked section of the root. These coordinates were then used to calculate the HGI and VGI as previously described (Grabov *et al*., 2004; Vaughn and Masson, 2011). Waviness was measured as the ratio of the cord to the root length (Grabov *et al*., 2004; Vaughn and Masson, 2011).

### GR24_rac_ and karrikins

Plants were grown for 6 days on the surface of control medium [0.8% (w/v) agar supplemented with ½ MS, including vitamins, pH 5.6], then transferred to medium containing racemic GR24_rac_ (LeadGen Labs, Orange, CT, USA), KAR_2_ or KAR_1_ (Toronto Research Chemicals, North York, ON, Canada), or only the carrier for the test compound as a control [sterile distilled water for KAR_2_ and KAR_1_, and 0.02% (v/v) acetone for GR24_rac_]. Plants were then grown for a further 3 days before scanning.

### Cell file rotation and root diameter analysis

Images of the root tips from plants grown vertically for 6 days, then placed at a 45° angle from the vertical for a further 3 days, were taken using a Leica DFC365FX camera attached to a Leica M205FA stereo microscope (Leica Microsystems, Cambridge, UK) with a Planapo × 1.6 objective set to magnification of × 80.5. Images were stitched using the LAS X software platform (Leica Microsystems). Following Wang *et al*. (2011), CFR was defined as the number of epidermal cell files that crossed a 1‐mm‐long straight line drawn down the longitudinal axis of the root from 1.5 to 2.5 mm from the root apex. Using the same images as for CFR measurements, root diameter was measured approximately 2 mm from the root apex using ImageJ (Schneider *et al*., 2012), three measurements were done per individual root.

### Mechanical stimulation assays for transcriptional response

Plants grown vertically on the surface of control plates for 9 days were transferred to a sterile buffer solution (0.1 mm KCl, 10 mm CaCl_2_ and 2 mm bis‐Tris propane, pH 5.8, adjusted with 0.5 m MES). A total of 30–40 seedlings per genotype were transferred into a Petri dish (3 cm in diameter), containing 3 ml of buffer solution, and left to acclimatize on the bench for 3 h with additional light (15W/865 Lumilux Daylight, maximum intensity: 86 μmol m^−2 ^sec^−1^). Mechanical stimulation was applied by shaking vigorously for 30 sec, while control plants remained on the bench. Plants were then left untouched for a further 30 min after stimulation before being immersed in RNALater (Sigma Aldrich, Gillingham, UK) for sample collection as described previously. For both assays, RNA was extracted from roots using the RNeasy Plant Mini kit (Qiagen, Manchester, UK) per manufacturer's instructions, including an additional DNase digestion step. A LiCl precipitation step was used to purify and concentrate the RNA before downstream quantitative polymerase chain reaction (qPCR) analysis.

### cDNA synthesis and transcript abundance measurement

Complementary DNA (cDNA) was synthesized from 500 ng RNA using the RT QuantiTect reverse transcription kit (Qiagen), following manufacturer's instructions, except that incubation time was lengthened for the gDNA Wipeout step (3 min at 42°C) and the cDNA synthesis (25 min at 42°C). cDNA was used as template in a quantitative real‐time PCR using the SYBR GREEN PCR kit (Qiagen) and the Rotor‐Gene 3000 thermocycler (Qiagen) to determine transcript abundance of the genes of interest *Calmodulin‐like* (*CML*) *12* and *CML24*. qPCR amplification cycle consisted of 5 min at 95°C followed by 40 cycles of 5 sec at 95°C and 10 sec at 60°C. Melting curves (ramping from 55°C to 95°C rising 1°C each step, with a 5‐sec delay between steps) were checked for unspecific amplification. qPCR traces were analysed using the R qpcR package (relevant parameters: data were normalised and the background subtracted; starting fit model: l4; efficiency estimation: cpD2; refmean: True; baseline subtraction using the average of the first five cycles; Ritz and Spiess, 2008; R package version 1.4‐0. 2015) to calculate Ct values. Efficiencies (all > 92%) were calculated using the calibration curve method. For each gene, the expression was calculated following the formula E = (eff^−Ct^). Expression of the genes of interest was normalised against two housekeeping genes *Ubiquitin 10* (*UBQ10*) and *Tubulin 4* (*TUB4*), as followed R_Gene of Interest_ **=* *E_Gene of Interest_/(sqrt(E_UBQ10_* E_TUB4_)). qPCR primers are listed in Table S1.

### Measurements of cytosolic Ca^2+^ concentration ([Ca^2+^]_cyt_) in response to mechanical stimulation

Col‐0 and *max2* [transformed using floral dip with *Agrobacterium tumefaciens* to express (apo)aequorin under a 35S promoter; Dodd *et al*., 2006] were used at T3 or T4 generation to determine cytosolic free Ca^2+^ concentration ([Ca^2+^]_cyt_). Equivalence of aequorin levels were determined by discharge assay of luminescence (> 4 million luminescence counts for both Col‐0 and *max2*). Plants were grown vertically on solid medium for 7–8 days as described above. Excised root tips (1 cm) were placed in the wells (one root per well) of a white 96‐well plate (Greiner Bio‐One, Stonehouse, UK) and incubated in 100 μl of bathing solution (10 μm coelentrazine; Lux Biotechnology, Edinburgh, UK 0.1 mm KCl, 10 mm CaCl_2_ and 2 mm bis‐Tris propane, pH 5.8 adjusted with 0.5 m MES) for 2 h in the dark, at room temperature. Luminescence was then recorded every second in a plate‐reading luminometer (FLUOstar Optima, BMG labtech, Ortenberg, Germany). After 35 sec, 100 μl of bathing solution (without coelentrazine) was injected into the well at 200 μl sec^−1^ to cause a mechanical stimulus to the root resulting in a sudden increase in luminescence (‘touch response’). The signal was monitored for a further 120 sec, when 100 μl of discharge solution [3 m CaCl_2_, in 30% (v/v) ethanol] was delivered to normalize the luminescence data and calculate [Ca^2+^]_cyt_ (Laohavisit *et al*., 2012). The [Ca^2+^]_cyt_ touch response of Col‐0 and *max2* were then compared.

### Root gravitropism assays

Arabidopsis plants were grown vertically for 14 days on the surface of control medium. On the day of the experiment, roots were positioned by aligning their root tips so that they could be imaged together. Plates were then placed vertically in the growth incubator but rotated through a 90° angle, thus inducing a 90° change in gravitropic orientation. Root tips were imaged using a Raspberry Pi camera module (http://www.raspberrypi.org/). Images were acquired every 10 min for 10 h. Image analysis was conducted using ARTT (Russino *et al*., 2013), which tracked the root tip growth and gave the tip orientation and displacement as output. Tip orientation was normalised to the displacement to take into account differences in growth rate.

### Data representation and statistical analysis

Root‐skewing data were represented using beanplots constructed in the R environment (R Core Team, 2012) using the beanplot package (Kampstra, 2014), to show the variability in root‐skewing angle. Statistical analyses were also conducted in the R environment. Normal distribution of the data and equality of variance were verified using Shapiro and Levene tests (Lawstat package; Gastwirth *et al*., 2017), respectively. Significant differences amongst genotypes were verified using one‐way anova, followed by Tukey HSD. anovas were conducted on rank values as a non‐parametric method, when data did not uphold the assumptions of normality and homoscedasticity. All experiments were repeated at least three times.

## Authors’ contribution

SMS and JMD planned and designed the research. SMS, YG, EM and FJ performed experiments and analysed data. SMS and JMD wrote the manuscript.

## Conflict of interest

The authors declare no conflicts of interest.

## Supporting information


**Figure S1.** Root‐skewing phenotype of *kai2‐2* mutant in the Col‐0 background, complemented mutants and *dlk2* mutants.

**Figure S2.** Effect of KAR on root skewing and primary root elongation in L*er*,* kai2* and *max2*.

**Figure S3.** Effect of GR24 on root skewing in *kai2*,* max2* and *d14*.

**Figure S4.** Gravitropic response of *max2*,* max3* and *max4* is faster than wild‐type.

**Figure S5.** *sks3* and *sku5* do not suppress the high LRD in *max2*.

**Figure S6.** *SKS3* and *SKU5* transcript cellular localisation in the root.

**Figure S7.** *MAX2* and *KAI2* transcript cellular localisation in the root.

**Table S1.** Primer sequences used in qPCR analysis.
