# Supplementary material for: Impairment in karrikin but not strigolactone sensing enhances root skewing in Arabidopsis thaliana
Source: Plant J. 2019 Mar 11;98(4):607–21. doi: 10.1111/tpj.14233 (PMC6563046; doi:10.1111/tpj.14233)
Supplement: Supplementary file 8 — Table S1. Primer sequences used in qPCR analysis. [file TPJ-98-607-s008.docx]

**Table S1:** Primer sequences used in qPCR analysis.

| **Gene** | **Direction** | **Sequence** |
| --- | --- | --- |
| *CML12* | Forward | 5'-AAGCCTTCCGCGTATTCGACAAGAA-3’ |
| *CML12* | Reverse | 5'-CACAAACTCAGAGAAACTGATGGTTCC-3’ |
| *CML24* | Forward | 5'-GAGTAATGGTGGTGGTGCTTGA-3’ |
| *CML24* | Reverse | 5'-ACGAATCATCACCGTCGACTAA-3’ |
| *UBQ10* | Forward | 5'-CCGACTACAACATTCAGAAGGA-3’ |
| *UBQ10* | Reverse | 5'-TCAGAACTCTCCACCTCCAAA-3’ |
| *TUB4* | Forward | 5'-CTGTTTCCGTACCCTCAAGC-3’ |
| *TUB4* | Reverse | 5'-AGGGAAACGAAGACAGCAAG-3’ |
