# Supplementary material for: Impairment in karrikin but not strigolactone sensing enhances root skewing in Arabidopsis thaliana
Source: Plant J. 2019 Mar 11;98(4):607–21. doi: 10.1111/tpj.14233 (PMC6563046; doi:10.1111/tpj.14233)
Supplement: Supplementary file 9 [file TPJ-98-607-s009.docx]

**Fig. S1.** **Root skewing phenotype of *kai2-2* mutant in the Col-0 background, complemented mutants and *dlk2* mutants.**

**(a)** The *kai2-2* mutant in the Col-0 background also displayed a significant increase in rightward root skewing.

**(b)** The root skewing angle of complemented lines of the *kai2-2* mutant was reduced compared to the *kai2-2* mutant but remained higher than that of the L*er* wild type. 10G and 12H are *kai2-2* lines complemented by *KAI2* expression under the native promoter. Data for each genotype are displayed as a beanplot with the skewing angle of individual roots shown as dark green horizontal lines, while the mean is represented by a thick black horizontal line. The estimated density of the distribution is illustrated by the shaded colour. The dashed line corresponds to the mean for the wild type. Significant differences compared to wild type (Tukey HSD) are shown * for *p* < 0.05 and ** for *p* < 0.01. Significant differences compared to *kai2-2* (Tukey HSD) are shown for *p* < 0.05 ‡ and *p* < 0.01 ‡‡. For each genotype, *n* > 48 in 4 separate experiments. **(c)** The root skewing angle of seedlings for three mutant alleles of *dlk2* showed no further increased compared to wild type. There is no significant difference between root skewing angle of *dlk2* alleles and wild type (Tukey HSD, *n.s.*). For each genotype, *n* > 98 in 4 separate experiments.

**Fig. S2**. **Effect of KAR on root skewing and primary root elongation in L*er*, *kai2* and *max2*.**

Root skewing angle of L*er* (**a**) and *kai2-2* (**c**) plants grown under control conditions or in the presence of 2.5, 5 or 10 µM KAR_2_ in the medium and, *max2-8* (**e**) grown under control conditions or in the presence of 5 µM KAR_2_ in the medium. Root elongation over a three-day period when L*er* (**b**), *kai2-2* (**d**) and *max2-8* (**f**) plants were exposed to KAR_2_. (**g**) Root skewing angle of L*er*, *kai2-2* and *max2-8* in the presence of 2.5, 5 and 10µM KAR_1_. Data for each genotype are displayed as a beanplot with the skewing angle of individual roots shown as dark green (or purple for the root elongation data) horizontal lines, while the mean is represented by a thick black horizontal line. The estimated density of the distribution is illustrated by the shaded colour. The dashed line corresponds to the mean for the control conditions. Significant differences compared to control conditions (Tukey HSD) are shown * for *p* < 0.05 and ** for *p* < 0.01. For each treatment and genotype combination, *n* > 64 (except for *kai2-2* under 2.5 and 10 µM where *n* > 30) in at least 3 independent experiments.

**Fig. S3. Effect of GR24 on root skewing in *kai2*, *max2* and *d14*.**

Root skewing angle of L*er* (**a**), *kai2-1* (**a**), *kai2-2* (**a**), Col-0 (**b**), *max2-1* (**b**), *d14* (**b**), *max3* (**d**) and *max4* (**e**) plants grown under control conditions or in the presence of 1 or 5 µM GR24 in the medium. Data for each genotype are displayed as a beanplot with the skewing angle of individual roots shown as dark green horizontal lines, while the mean is represented by a thick black horizontal line. The estimated density of the distribution is illustrated by the shaded colour. The dashed line corresponds to the mean for the control conditions. * indicates a significant difference compared to control conditions (Tukey HSD, *p* < 0.05). For each treatment and genotype combination, *n* > 75 in at least 3 independent experiments.

**Fig. S4.** **Gravitropic response of *max2*, *max3*, and *max4* is faster than wild type.**

The tip orientation of roots from wild type (Col-0), *max2, max3* and *max4* was recorded every 10 min and for 10 h after a change in gravitropic orientation. The change in tip orientation was normalised to the tip displacement to account for differences in growth rate between genotype. Data are shown as mean ± se, *n* = 6-13 plants per genotypes, obtained in at least 3 experiments.

**Fig. S5. *sks3* and *sku5* do not suppress the high lateral root density in *max2.***

**(a)**. Lateral roots per cm of primary roots in 9-d-old seedlings. Data are shown as mean ± se. For each genotype, n > 51 plants grown in 5 separate experiment. Letters indicate significant differences (Tukey HSD, *p* < 0.05). **(b)**. *sks3* does not suppress low germination rate in *max2*

Seeds were germinated on 0.8% (w/v) agar plates and germination rate was scored after 72h. Data are shown as mean ± se, for 10 batches of seeds each batch holding > 80 seeds. Significant differences compared to wild type (Tukey HSD) are shown as • for *p* < 0.1, * for *p* < 0.05 and ** for *p* < 0.01.

**Fig. S6.** ***SKS3* and *SKU5* transcript cellular localisation in the root.** (**a)** and **(d)**, absolute transcripts abundance of *SKS3* (At5g48450) and *SKU5* (At4g12420) in the root, obtained from the eFP browser (http://bar.utoronto.ca/efp/cgi-bin/efpWeb.cgi, Brady et al. 2007; Winter et al., 2007). **(b)** and **(e)** colour scale for expression levels. **(c)** and **(f)** histogram of average expression level of all genes in the root data set. The red line indicates the maximum expression for *SKS3* (**c**) and for *SKU5* (**f**).

**Fig. S7.** ***MAX2* and *KAI2* transcript cellular localisation in the root.** **A** and **D**, absolute transcripts abundance of *MAX2* (At2g42620) and *KAI2* (At4g37470) in the root, obtained from the eFP browser (http://bar.utoronto.ca/efp/cgi-bin/efpWeb.cgi, Brady et al. 2007; Winter et al., 2007). **(b)** and **(e)** colour scale for expression levels. **(c)** and **(f)** histogram of average expression level of all genes in the root data set. The red line indicates the maximum expression for *MAX2* (**c**) and for *KAI2* (**f**).

**Table S1:** Primer sequences used in qPCR analysis.
